# Comparison of temporomandibular joints in relation to ages and vertical facial types in skeletal class II female patients: a multiple-cross-sectional study

**DOI:** 10.1186/s12903-024-04219-4

**Published:** 2024-04-17

**Authors:** Jing Zhou, Huifang Yang, Qing Li, Weiran Li, Yi Liu

**Affiliations:** 1 Department of Orthodontics, Peking University School and Hospital of Stomatology & National Center for Stomatology & National Clinical Research Center for Oral Diseases, No.22 Zhongguancun South Avenue, Haidian District, Beijing, 100081 China; 2 National Engineering Research Center of Oral Biomaterials and Digital Medical Devices & NHC Key Laboratory of Digital Stomatology, Beijing Key Laboratory of Digital Stomatology, Peking University School and Hospital of Stomatology & National Center for Stomatology & National Clinical Research Center for Oral Diseases, Beijing, 100081 China

**Keywords:** Temporomandibular joint, Skeletal pattern, Adolescent, Adult, CBCT

## Abstract

**Background:**

The temporomandibular joint (TMJ) is closely related to the dynamic balance and stability of mandibular function and orthodontic treatment. Skeletal class II female patients are thought to be at high risk for TMJ disease. The relationship between the TMJ and craniofacial structures is still controversial. This study compared the morphology and position of the TMJ in skeletal class II adolescents and adults with various vertical facial types using cone-beam computed tomography (CBCT).

**Materials and methods:**

A total of 117 skeletal class II patients were divided into three groups according to the FH-GoGn angle (hypodivergent, normodivergent and hyperdivergent), with 40 class I normodivergent patients serving as controls. Each group contained two age subgroups (adolescents: 11–14 years old, adults: 18–35 years old). The size (condylar length, height, long and short axis diameter, glenoid fossa width and depth) and shape (condylar neck inclination, condylar head angle and long axis angle, articular eminence inclination) of the condyle and fossa, joint space (anterior, superior, posterior, mesial and lateral), and position of the fossa (vertical, transverse, and anteroposterior distance) and condyle were measured and compared using CBCT.

**Results:**

Class II hypodivergent patients exhibited the greatest condylar length, height, and long- and short-axis diameter; steepest articular eminence; deepest fossa depth; largest superior, mesial and lateral joint spaces; and highest fossa position in both age groups. The manifestations of class II hyperdivergent patients were mostly the opposite. In adults, except for the condylar long axis angle, the measurements of the condyle increased differently among skeletal patterns, while the measurements of the fossa decreased, as the joint spaces and fossa position remained approximately stable compared with those in adolescents.

**Conclusion:**

The vertical skeletal pattern, rather than the class II sagittal skeletal pattern, may be the main factor affecting the morphology and position of the TMJ. Attention should be given to the TMJ area in hyperdivergent patients with a relatively poor-fit condyle-fossa relationship. The changes in the TMJ with age were mainly morphological rather than positional and varied with skeletal pattern.

## Background

The temporomandibular joint (TMJ) is closely related to the dynamic balance of mandibular function and plays a crucial role in chewing, swallowing, breathing and language [1]. The anatomical structure as well as the position of the condyle and fossa may play important predictive roles in accurately identifying degenerative changes in the TMJ [2] and affecting the long-term stability of orthodontic and orthognathic treatment [3–5].

Skeletal class II malocclusion is the most common orthodontic problem, occurring in approximately 1/3 of all orthodontic populations [6]. The incidence of temporomandibular joint disease (TMD), which influences the effectiveness and stability of orthodontic treatment [7], is the highest in patients with skeletal class II facial patterns [8].

An association between the TMJ and craniofacial structure is suspected [9], although there is some controversy [10, 11]. On the one hand, as one of the main components, the condyle is an important growth site of the mandible [12]. The vertical growth of the condyle, together with the descending glenoid fossa, determines the position of the mandible and ultimately affects the facial type [13]. On the other hand, the TMJ retains the capacity for lifelong remodelling in response to external stimulation [14]. The maximum bite force and masticatory muscle function change with craniofacial morphology [15], generating different functional environments [16, 17] and resulting in adaptive TMJ remodelling and morphological variation. Most studies have shown that TMJ morphology and condylar position are affected by skeletal facial type [9], although the actual effect remains controversial [18]. These studies mostly involved only one age group [3, 4, 9, 19–25], which necessarily could not show dynamic changes in the TMJ with age, divided only by sagittal [4, 9, 10, 21, 22, 26, 27] or vertical skeletal patterns [4, 9, 10, 20, 24, 26], which probably increased potential confounding factors. To our knowledge, although some studies have taken age into account [10, 28], the differential changes in the TMJ with age in skeletal class II patients with different vertical facial types remain unclear. The aim of this study was to use cone-beam computed tomography (CBCT) to compare the TMJ morphology and position of skeletal class II female patients according to vertical facial type, including both adolescents and adults, to explore changes in the TMJ during mid-to-late puberty and provide a reference for orthodontic and orthognathic clinical use.

## Materials and methods

This study was approved by the human subjects ethics board of Peking University School and Hospital of Stomatology Research Ethical Committee (PKUSSIRB-202054053) and was conducted in accordance with the Helsinki Declaration of 1975, as revised in 2013 [29].

The sample size was calculated on the basis of data from published literature [30] through the One-way Analysis of Variance module in PASS software (version 21.0, NCSS, Kaysville, US) with α,number of groups and power values set at 0.05, 4 and 90%. The hypothesized means were set at 8.630, 9.717, 9.480, and 8.157, while the standard deviation of subjects were set at 1.227, 1.432, 1.277, 1.569. Twenty-three condyles (i.e., twelve patients) per group were needed. According to previous studies [19, 23, 24, 31], we included 20 patients/group, except for the class II hyperdivergent adult group since only 17 patients met the inclusion criteria.

The sample consisted of 157 patients, including 80 adolescents and 77 adults, who had undergone craniofacial CBCT scans at the Peking University School and Hospital of Stomatology for orthodontic treatment from February 2015 to June 2020. Patients were selected according to the following inclusion criteria: (1) female, aged 11–14 years for adolescents or 18–35 years for adults; (2) ANB angle ≥0°; (3) no facial asymmetry (menton deviation less than 3 mm from the midsagittal plane); and (4) fully erupted permanent teeth, or permanent teeth congenitally missing or impacted with corresponding normal functioning deciduous teeth retained. The exclusion criteria were as follows: (1) history of cleft lip or palate, craniofacial syndrome, trauma, orthodontic treatment, or surgery; (2) crowns, implants, or extensive decay or filling of teeth; (3) traumatic occlusion, including open bite, scissors bite, and closed deep overbite; (4) alveolar bone resorption exceeding 1/3 of root length; (5) TMD symptoms, including pain, discomfort, restricted mouth opening, or visible bone changes on imaging of the TMJ area; and (6) oral parafunctions such as bruxism.

A total of 117 skeletal class II patients were divided into three groups according to the FH-GoGn angle, with 40 class I normodivergent patients serving as controls. Each group included two age subgroups (Table 1).

**Table 1:** Grouping criteria based on age and cephalometrics

Skeletal pattern	ANB(°)	FH-GoGn(°)	Age subgroups
Control group	0° ≤ ANB<4°	22° ≤ FH-GoGn≤32°	Group C1: adolescent
			Group C2: adult
Class II hypodivergent	ANB ≥ 4°	FH-GoGn<22°	Group L1: adolescent
			Group L2: adult
Class II normodivergent		22° ≤ FH-GoGn≤32°	Group N1: adolescent
			Group N2: adult
Class II hyperdivergent		FH-GoGn>32°	Group H1: adolescent
			Group H2: adult

**Fig. 1:**
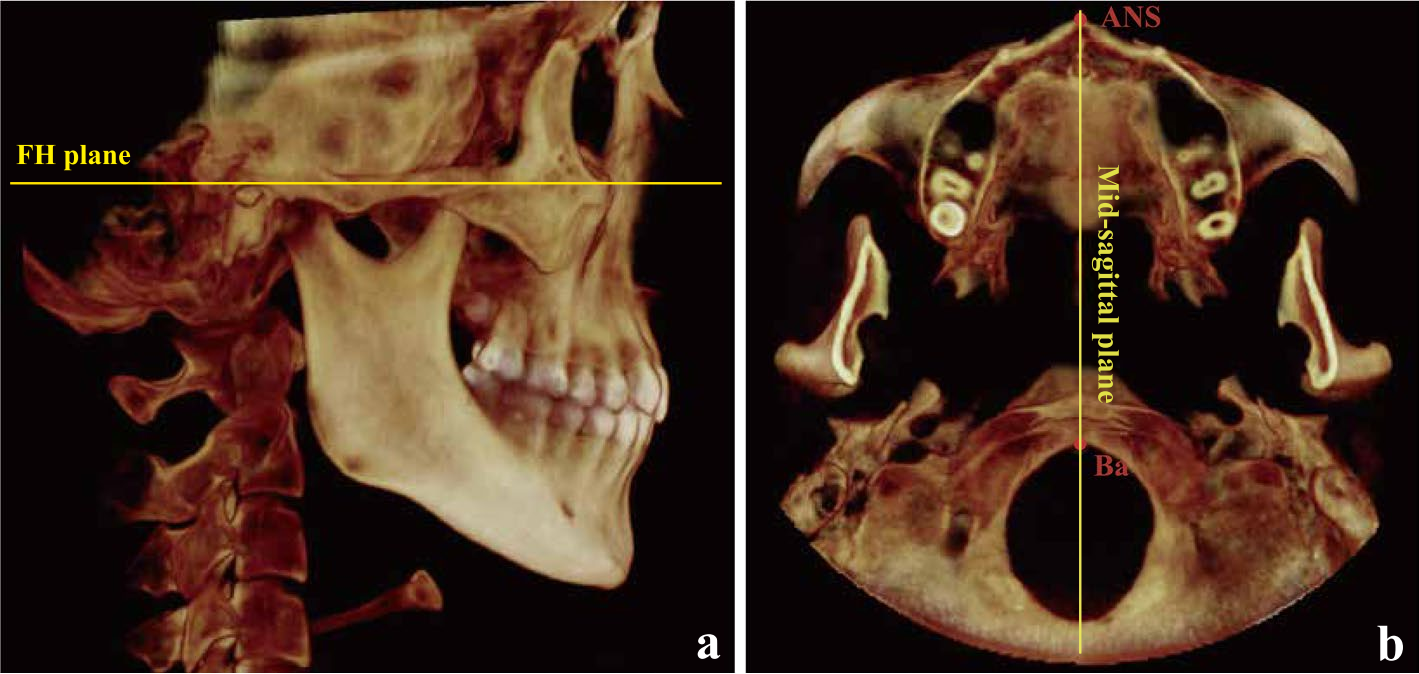
**a** FH plane parallel to the horizontal plane. **b** Midsagittal plane passing through the ANS point and Ba point

The CBCT equipment used was a NewTom system (NewTom VG, Volumetric Scanner, Aperio, Italy), and the images were obtained at 110 kV, 3.5 mA, an exposure time of 3.6 seconds, a field of view of 15 × 15 cm and a voxel size of 0.3 mm. The patients were instructed to stand upright, breath steadily, bite with intercuspal occlusion (ICO), and look forward to maintain the Frankfort horizontal (FH) plane parallel to the floor with a headband and chin support. Images were saved as Digital Imaging and Communication in Medicine (DICOM) files and reconstructed in Dolphin software (version 11.9, Dolphin Imaging and Management Solutions, Chatsworth, CA). Reorientation was performed so that the bilateral FH plane was parallel to the horizontal plane (Fig. 1a), and the midsagittal plane passed through the skull base point (Ba) and anterior nasal spine (ANS) point (Fig. 1b) at the same time.

**Fig. 2:**
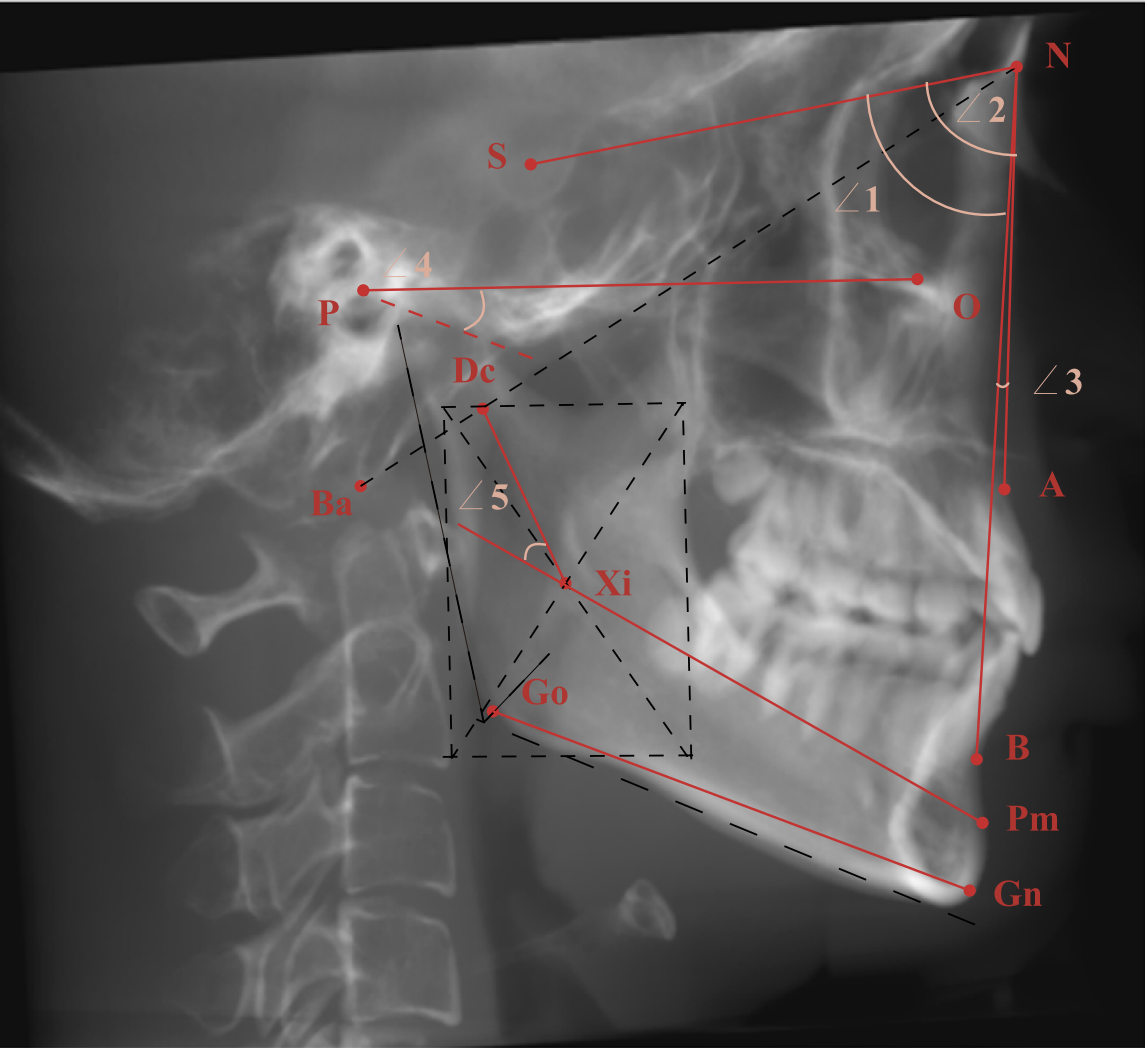
Construction of landmarks used in the cephalometric analysis and angular measurements. (**∠**1: SNB ∠2: SNA ∠3: ANB ∠4: FH-GoGn ∠5: mandibular arc)

**Fig. 3:**
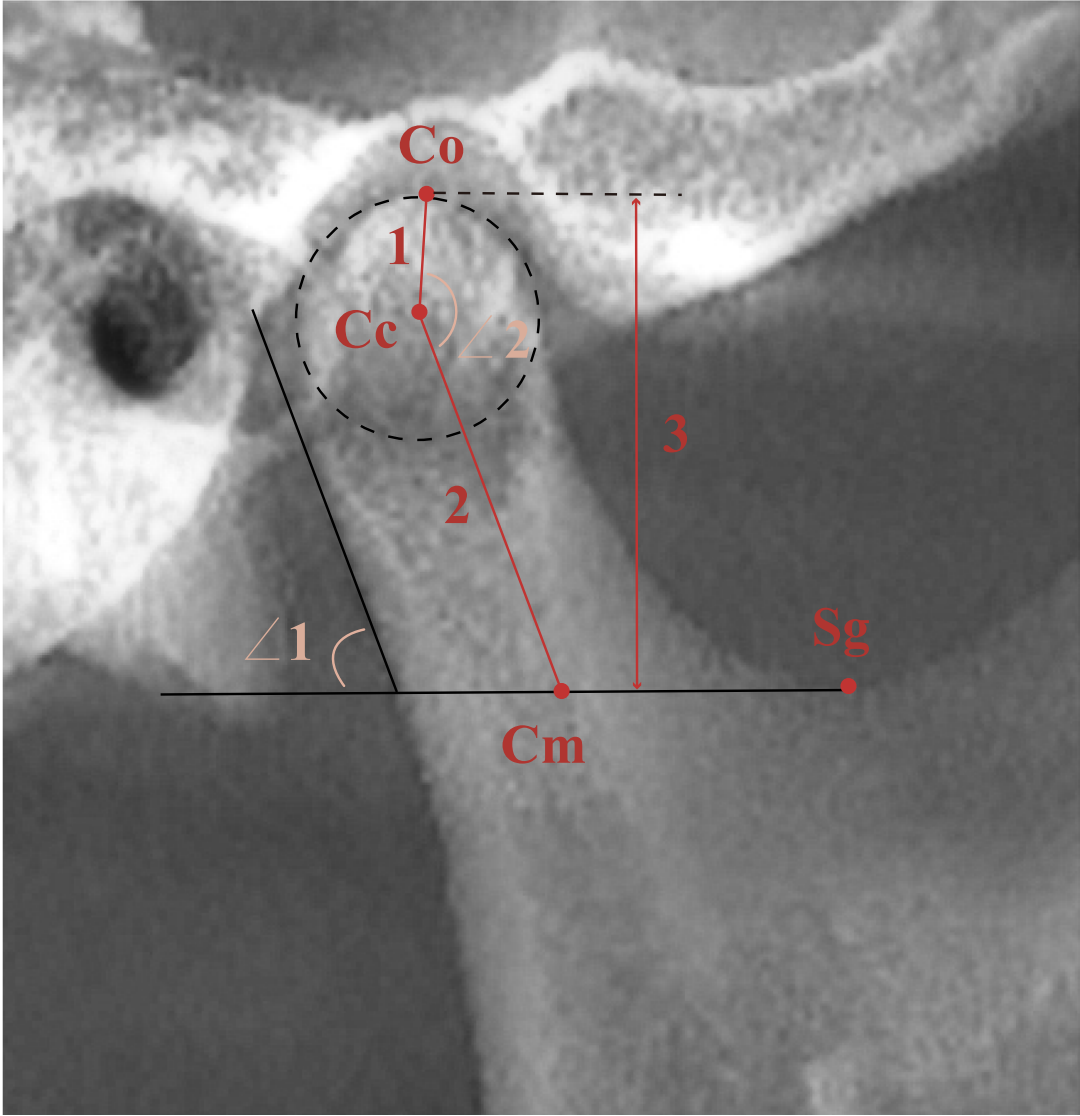
Landmarks and measurements of the sagittal projection of the mandibular ramus (Co: superior point of the condyle; Cc: centre of the largest circle that fits the condylar head arc; Sg: inferior point of the mandibular sigmoid incisure; Cm: intersection of the horizontal line passing through Sg and line 2, which passes through Cc parallel to the tangent of the condylar posterior border; ∠1 Cni: condylar neck inclination, which is the posterior superior angle between the tangent line of the condyle posterior border and a horizontal line; ∠2 Condylar head angle: angle between the condylar head and neck, anterior angle between line 1 and line 2; Line 1: distance between Co and Cc; Line 2: distance between Cc and Cm; Condylar length: line 1 plus line 2; Line 3: condylar height: vertical distance between Co and Sg)

**Fig. 4:**
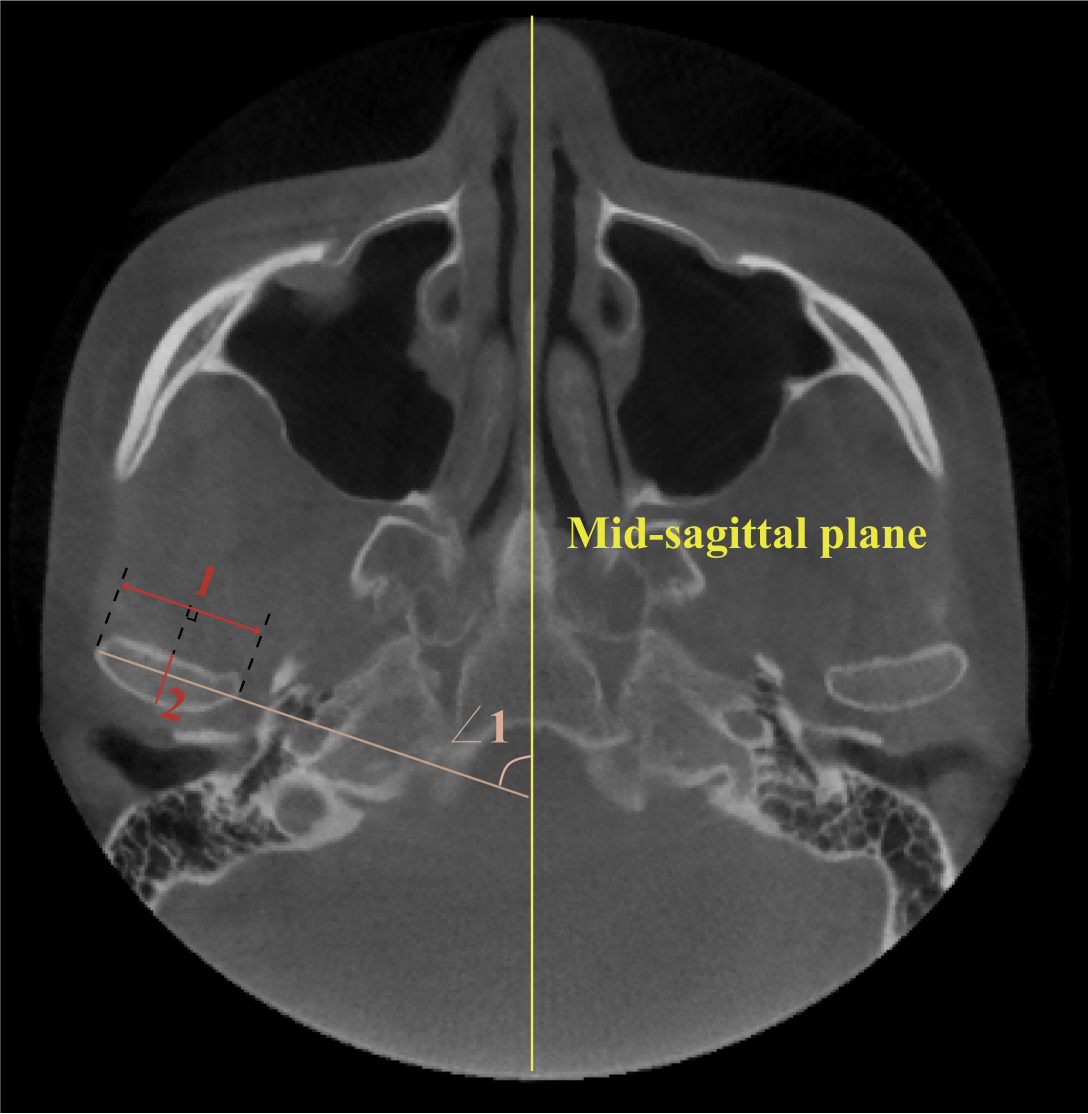
Measurements on the largest axial view of the condyle. (∠1 Condylar long axis angle: angle between the condylar mediolateral axis and the midsagittal plane; 1: Condylar long axis diameter: largest mediolateral diameter of the condyle; 2: Condylar short axis diameter: largest anteroposterior diameter of the condyle, perpendicular to line 1)

**Fig. 5:**
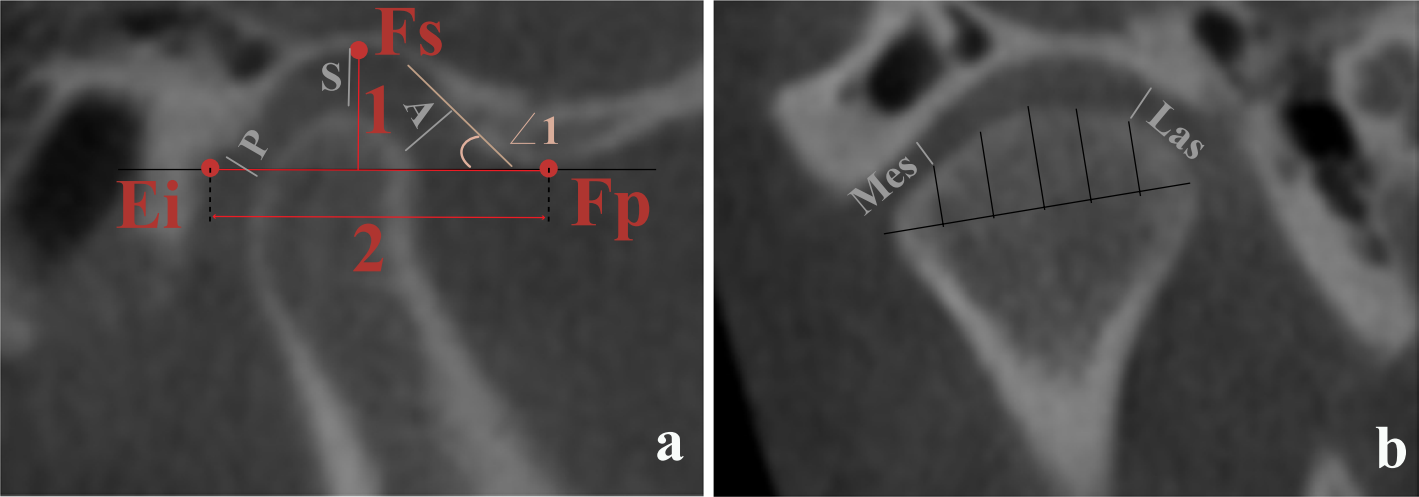
**a** Midsagittal plane of the condyle. (Ei: inferior point of the articular eminence; Fs: superior point of the glenoid fossa; Fp: intersection of the posterior slope of the glenoid fossa and a line parallel to the FH plane and passing through the Ei. When the posterior slope was shorter than the anterior slope, the inferior point was used. ∠1 Aei: articular eminence inclination, the angle between the best fit line of the posterior slope of the articular eminence and the horizontal plane. 1: Glenoid fossa depth; 2: Glenoid fossa width. A: Anterior joint space: shortest distance from the most prominent anterior point of the condyle to the corresponding glenoid fossa bone. S: Superior joint space: shortest distance from the most superior point of the condyle to the Fs. P: Posterior joint space: shortest distance from the most prominent posterior point of the condyle to the corresponding glenoid fossa bone.) **b** Midcoronal plane of the condyle (Mes: the medial joint space, the shortest distance from the midpoint between the most medial and superior points of the condyle to the corresponding glenoid fossa bone. Las: lateral joint space, the shortest distance from the midpoint of the most lateral and superior points of the condyle to the corresponding glenoid fossa bone

**Fig. 6:**
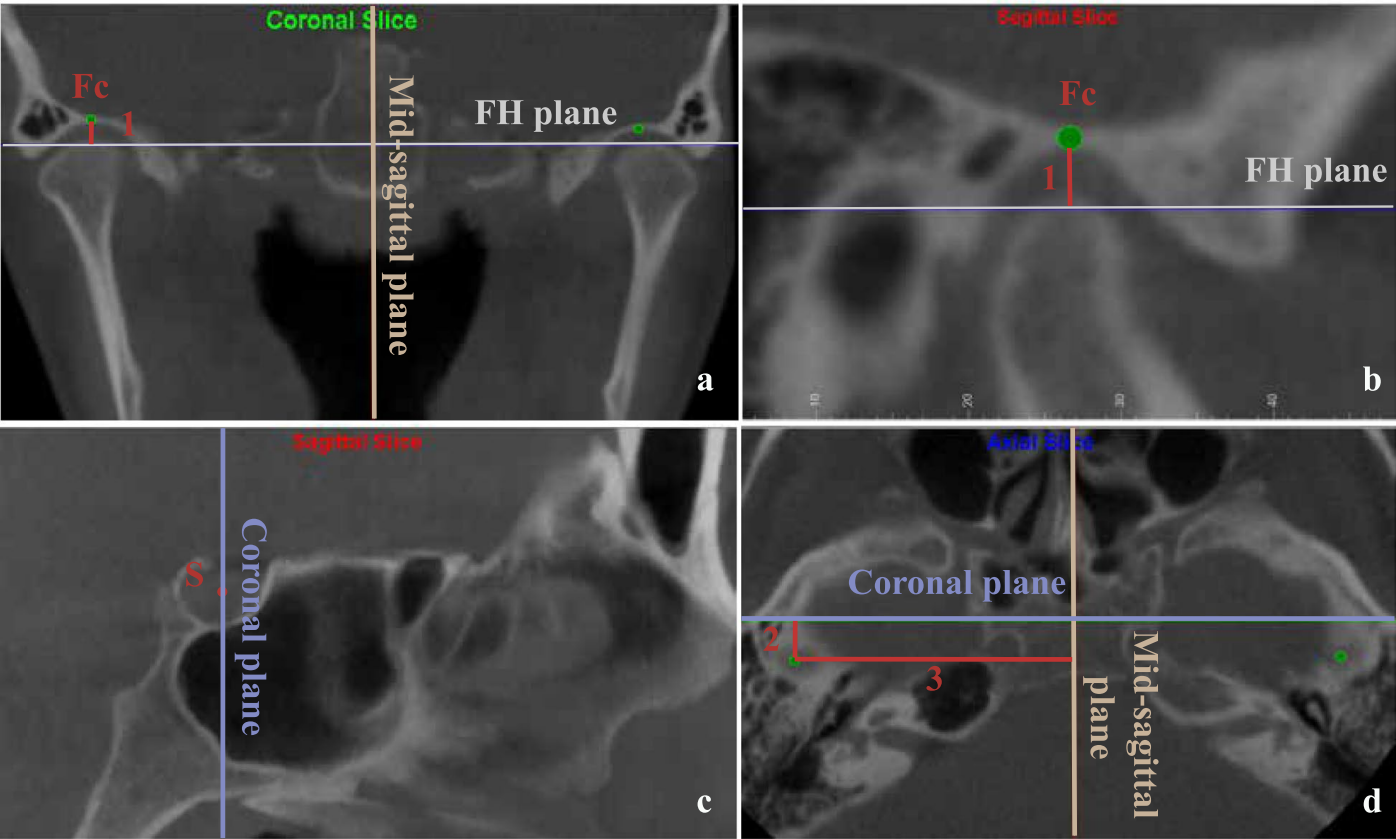
**a**,** b** Location of the glenoid fossa centre point and its vertical distance measurement (Fc: glenoid fossa centre point, the most superior point of the glenoid fossa on the sagittal and coronal planes. 1: Glenoid fossa vertical distance, the vertical distance between the Fc point and the FH plane; positive when Fc is higher); c Sella point located on the midsagittal plane, with the coronal plane orientated through it; Fig. 6d Axial view (2: Glenoid fossa sagittal distance, sagittal projection distance from the Fc to S. 3: Glenoid fossa coronal distance, distance from the Fc to the midsagittal plane)

Lateral cephalometric images were obtained by Dolphin software using the parallel projection method with the direction from right to left (Fig. 2). Four planes were used in the measurements, and their definitions are illustrated in Table 2 and shown in Figs. 3, 4 and 5. The landmarks and measurements used for analysis are presented in Figs. 2, 3, 4 and 6. Using the formula ln(P/A) [32], where A represents the anterior joint space and P represents the posterior joint space, the relative position of the condyle in the glenoid fossa was determined. The condyle was defined as posterior if the ratio was less than − 0.25, anterior if the ratio was greater than + 0.25, or concentric when the ratio was within ±0.25.

**Table 2:** Definitions of the measurement planes

Measurement planes	Definition
Sagittal projection plane of mandibular ramus (Fig. 3)	In the cranial lateral view, intercept both sides of mandibular ramus separately.
Maximum axial plane of condyle (Fig. 4)	In the joint view, setting the layer to 0.5 mm thickness and 40.0 mm long, define the section of condyle layer at maximum mediolateral diameter.
Midsagittal plane of condyle (Fig. 5a)	the section perpendicular to the maximum axial plane of the condyle and vertically passing through the midpoint of mediolateral diameter.
Midcoronal plane of condyle (Fig. 5b)	the section perpendicular to the maximum axial plane of the condyle and parallel to the maximum mediolateral diameter.

Patients were numbered to blind the investigators. One investigator (Dr Zhou) performed all the measurements using Dolphin 11.9 software (Dolphin Imaging and Management Solutions, Chatsworth, California) on the same 14-in. monitor computer (Lenovo, ThinkPad, L440) with a resolution of 1600 × 900 pixels over 2 weeks. The image evaluations were conducted in a separate quiet space with adequate light. The investigator took a 15-minute break for every hour of measurement.

### Statistical analysis

SPSS software (version 24.0 for Windows; IBM, Chicago, US) was used for the statistical analysis. The Shapiro–Wilk normality test was performed. Depending on whether normality was satisfied, an independent sample t test or Wilcoxon signed-rank test was used to compare the TMJ measurements between age groups with the same skeletal pattern. One-way analysis of variance (ANOVA) was used to compare the mean values of baseline measurements (including age, ANB, and FH-GoGn) and TMJ measurements between vertical and horizontal skeletal patterns of the same age. The Kruskal–Wallis test was performed when the variance analysis did not satisfy normality and homogeneity. For pairwise comparisons, a least significant difference t test (LSD-t) was conducted when ANOVA was significant, and a Nemenyi test was used when the Kruskal–Wallis test was significant. The chi-square test or Fisher’s exact test was used for crosstabs. Significance was defined as *P* < 0.05. Post hoc Bonferroni correction was used to determine the significance for multiple comparisons (*P* < 0.0083).

## Results

### Intra- and inter-observer reliability

To test the reliability of the measurements, 20 CBCT images were randomly selected for remeasurement 2 weeks after the initial measurement by the same investigator (Dr. Zhou) and another investigator (Dr. Dong). The intraclass correlation coefficient (0.834–0.999 for Dr. Zhou) and interclass correlation coefficient (0.828–0.991) showed excellent intra- and inter-observer reliability.

### Baseline comparisons

There were no significant differences in age among participants (*P* > 0.05) in the same age subgroup, while ANB and FH-GoGn were significantly different (*P* < 0.01) (Table 3). In adolescents, the mean ANB value was significantly lower in the control group than in the other three groups (*P* < 0.001); the mean FH-GoGn value was the greatest in the class II hyperdivergent group (*P* < 0.001) and smallest in the class II hypodivergent group (*P* < 0.001), and it was almost equal between the control group and class II normodivergent group (*P* > 0.05). The results for the adults were similar to those for the adolescents.

**Table 3:** Ages and cephalometrics distribution of subjects among groups

Groups	Class I norm		Class II hypo		Class II norm		Class II hyper		*P* < 0.01	*P* < 0.0083
	Group C1	Group C2	Group L1	Group L2	Group N1	Group N2	Group H1	Group H2		
Age (month, mean ± SD)	154.20 ±10.07	289.10 ±66.46	152.55 ±12.70	328.35 ±62.33	153.00 ±11.73	295.85 ±61.65	151.65 ±11.47	296.47 ±63.76	C1 v C2, L1 v L2, N1 v N2, H1 v H2	
ANB (°, mean ± SD)	2.51 ±1.01	2.35 ±1.03	4.98 ±0.89	5.44 ±2.27	5.36 ±1.17	5.98 ±1.44	6.36 ±1.48	6.86 ±2.07		C1 v L1, N1, H1 C2 v L2, N2, H2
FH-GoGn (°, mean ± SD)	26.18 ±2.10	25.36 ±2.65	19.47 ±2.74	19.51 ±2.27	26.05 ±1.79	25.32 ±2.07	33.71 ±1.89	33.89 ±2.47		C1, N1 v L1, H1 C2, N2 v L2, H2

*SD* Standard deviation

The mean values showed no significant differences in the baseline measurements (P > 0.05) except for age (P < 0.001) between the two age subgroups with the same skeletal pattern (Table 3).

### Assessment of the TMJ according to class II sagittal skeletal patterns

TMJ measurements were compared between the control group and the class II normodivergent group (Table 4). There were no statistically significant differences in any of the TMJ morphological measurements between class I and II normodivergent adolescents. In adults, the condylar height of the class II normodivergent group was significantly smaller than that of the class I group, while the superior and lateral joint spaces were significantly greater. No other significant differences were observed.

**Table 4:** Comparison of TMJ measurements among groups(mm/°, mean ± standard deviation)

Measurements	Class I normodivergent		Class II hypodivergent		Class II normodivergent		Class II hyperdivergent		*P* < 0.05	*P* < 0.0083
	Group		Group		Group		Group			
	C1	C2	L1	L2	N1	N2	H1	H2		
Size of condyle and fossa										
Condylar length	17.30 ±1.92	19.49 ±2.13	17.16 ±2.43	19.58 ±2.50	17.32 ±2.78	18.27 ±3.11	17.39 ±1.77	18.38 ±2.38	C1 v C2 L1 v L2, H1 v H2	L2 v N2
Condylar height	16.48 ±1.76	18.74 ±2.01	16.39 ±2.42	18.85 ±2.39	16.55 ±2.73	17.52 ±2.99	16.63 ±1.76	17.66 ±2.29	C1 v C2 L1 v L2, H1 v H2	C2, L2 v N2
Condylar long axis diameter	16.99 ±2.61	18.15 ±1.96	17.37 ±1.76	19.12 ±2.49	16.94 ±2.00	18.00 ±2.16	15.54 ±2.51	16.67 ±2.14	L1 v L2 N1 v N2, H1 v H2	C1, L1 v H1 L2 v H2
Condylar short axis diameter	7.73 ±1.01	8.11 ±0.97	7.85 ±1.14	8.91 ±1.26	7.77 ±0.95	8.23 ±1.39	7.55 ±1.00	8.36 ±0.98	L1 v L2 H1 v H2	C2, N2 v L2
Glenoid fossa width	17.35 ±2.08	17.41 ±1.15	17.41 ±1.52	16.78 ±2.13	17.50 ±2.25	16.49 ±1.72	17.07 ±1.78	16.67 ±2.56	N1 v N2	
Glenoid fossa depth	6.62 ±1.33	6.69 ±1.39	7.37 ±1.03	6.86 ±1.04	6.96 ±1.01	6.83 ±1.21	6.50 ±1.15	6.08 ±1.03	L1 v L2	C1, H1 v L1 L2, N2 v H2
Shape of condyle and fossa										
Condylar neck inclination	73.48 ±6.07	74.24 ±4.38	73.23 ±6.01	75.50 ±4.86	73.35 ±5.08	75.35 ±5.52	73.99 ±6.47	76.04 ±4.98	C1 v C2	
Condylar head angle	151.56 ±7.96	160.40 ±6.74	154.12 ±13.95	160.93 ±5.76	152.44 ±10.15	161.39 ±8.91	154.26 ±10.15	161.47 ±8.31	L1 v L2 N1 v N2, H1 v H2	
Articular eminence inclination	49.62 ±11.76	41.94 ±9.47	53.24 ±9.09	47.50 ±8.00	50.42 ±8.77	46.75 ±8.32	49.43 ±11.34	42.12 ±8.06	C1 v C2 L1 v L2 H1 v H2	C2, H2 v L2
Condylar long axis angle	68.24 ±8.43	69.32 ±8.55	68.15 ±5.38	66.84 ±7.19	68.02 ±7.69	69.88 ±8.42	65.79 ±9.03	67.19 ±11.86		
Mandibular arc	31.41 ±4.47	33.27 ±4.20	35.71 ±3.54	39.26 ±4.97	31.60 ±4.03	34.94 ±4.85	24.30 ±3.22	28.11 ±3.91	L1 v L2 N1 v N2 H1 v H2	C1, L1, N1 v H1 C2, L2, N2 v H2
Joint space										
Anterior joint space	1.55 ±0.56	1.63 ±0.63	1.71 ±0.40	1.91 ±0.82	1.70 ±0.55	1.81 ±0.60	1.82 ±0.60	1.74 ±0.62		
Superior joint space	2.23 ±0.63	2.34 ±0.57	2.91 ±0.77	2.74 ±0.50	2.42 ±0.58	2.77 ±0.76	2.07 ±0.74	2.37 ±0.73	N1 v N2	C1, N1, H1 v L1 C2 v L2, N2
Posterior joint space	1.65 ±0.48	1.40 ±0.36	1.68 ±0.48	1.64 ±0.43	1.58 ±0.39	1.63 ±0.48	1.49 ±0.46	1.59 ±0.55	C1 v C2	
Mesial joint space	2.11 ±0.62	2.31 ±0.69	2.50 ±0.74	2.49 ±0.70	2.19 ±0.53	2.50 ±0.70	1.91 ±0.62	2.03 ±0.65	N1 v N2	L1 v H1 L2, N2 v H2
Lateral joint space	2.09 ±0.61	1.68 ±0.57	2.34 ±0.52	2.17 ±0.69	2.05 ±0.71	2.15 ±0.66	1.88 ±0.66	1.84 ±0.85	C1 v C2	L1 v H1 C2 v L2, N2
Fossa position										
Glenoid fossa vertical distance	−2.63 ±1.42	−2.10 ±1.52	−1.53 ±1.36	−1.54 ±1.35	−2.01 ±1.29	−1.95 ±1.36	−2.90 ±1.32	−2.64 ±1.78		L1 v C1, H1 H1 v L1, N1 L2 v H2
Glenoid fossa transverse distance	49.02 ±2.44	49.07 ±1.96	48.56 ±1.87	49.48 ±2.70	49.12 ±2.34	49.08 ±2.42	48.59 ±2.40	50.19 ±1.84	H1 v H2	
Glenoid fossa anteroposterior distance	9.65 ±3.12	9.75 ±2.21	9.60 ±3.11	8.95 ±2.21	10.38 ±2.13	8.90 ±2.09	9.67 ±2.76	8.84 ±2.77	N1 v N2	

*SD* Standard deviation

### Assessment of the TMJ according to different class II vertical skeletal patterns

TMJ measurements were compared between the control group and the class II groups with different vertical facial types (Table 4). No significant differences in condylar length or height were observed among the adolescents. In adults, condylar length and height were the greatest in the hypodivergent group, similar to those in the control group, and significantly differed from those in the normodivergent group (*P* < 0.0083), which were similar to those in the hyperdivergent group. The trends in the other TMJ measurements were similar for the two age groups: the class II hypodivergent patients exhibited the longest condylar long and short axes, largest mandibular arc, deepest glenoid fossa depth, steepest articular eminence inclination, and lowest glenoid fossa vertical position. The class II hyperdivergent patients exhibited almost opposite results. There were no significant differences in glenoid fossa width, condylar shape (including condylar neck inclination, condylar head angle or condylar long axis angle), anterior or posterior joint space, or glenoid fossa transverse or anteroposterior distance among skeletal class II vertical facial patterns in either age group. The shape, joint spaces and fossa position were significantly different (P < 0.0083), however, as the articular eminence inclination was significant only in adults.

### Assessment of the TMJ according to age with the same skeletal pattern

Comparisons between two age groups with the same skeletal pattern were made (Table 4). Regarding condyle and fossa size and shape, although not all differences between groups were statistically significant, the condylar length, height, long and short axis diameter, neck inclination, head angle and mandibular arc were greater in adults than in adolescents with all the skeletal patterns. The average differences between the two ages were greastest in hypodivergent patients, excluding in the condylar head angle and mandibular arc. The glenoid fossa width and depth were smaller in adults than in adolescents except for the control group, in which they were almost identical. The articular eminence inclinations for adults were also smaller in all the comparisons. No statistically significant differences were detected in the condylar long axis angle. Joint spaces were almost identical of the two ages in most groups except in the control group, which showed slightly smaller posterior and lateral joint spaces in adults, and in the class II normodivergent group, which showed slightly greater upper and mesial joint spaces (*P* < 0.05) in adults compared with those of adolescents. The range of these differences were within 0.4 mm. In terms of fossa position, there was no statistically significant difference between adults and adolescents, except for a slightly smaller anteroposterior distance of class II normodivergent adults (*P* < 0.05) and a slightly greater transverse distance of hyperdivergent adults (*P* < 0.05) compared with those of adolescents.

### Condyle position distribution

There were significant differences in the condyle sagittal position distribution computed by the formula ln(P/A) among different skeletal patterns in adolescents (Table 5, *P* < 0.05); the condyles of the control group were mainly in the anterior and concentric positions, while those in the class II groups were mainly in the anterior and posterior positions, with the proportion of posteriorly positioned condyles gradually increasing from hypodivergent to hyperdivergent. However, in adults, there was no significant difference (*P* > 0.05), although the proportion of posteriorly positioned condyles in hyperdivergent patients was the highest, while the proportions in the other three groups were similar. For patients with the same skeletal pattern, no significant difference in the condylar position distribution was observed between adults and adolescents except for hypodivergent patients (*P* < 0.05).

**Table 5:** Distribution of condylar position in each group (side (%))

Group		Condylar position		
		Anterior	Concentric	Posterior
Class I normodivergent	Group C1	11(27.5%)	22(55%)	7 (17.5%)
	Group C2	9 (22.5%)	16 (40%)	15 (37.5%)
Class II hypodivergent	Group L1	4 (10%)	25 (62.5%)	11(27.5%)
	Group L2	11(27.5%)	14 (35%)	15 (37.5%)
Class II normodivergent	Group N1	11(27.5%)	12 (30%)	17 (42.5%)
	Group N2	8 (20%)	17 (42.5%)	15 (37.5%)
Class II hyperdivergent	Group H1	6 (15%)	16 (40%)	18 (45%)
	Group H2	9 (26.5%)	10 (29.4%)	15 (44.1%)

## Discussion

The TMJ is essential for stable biting and chewing [31]. Its position and morphology are affected by many factors, such as sex, age, growth type, facial symmetry, functional movement, mechanical functional environment and intra-articular pathologies [18]. The relationship between craniofacial structure and the TMJ remains controversial [16, 19, 24]. It has been suggested that the condylar functional load during mastication is determined by craniofacial structure, and changes in the mechanical functional environment might affect the diversity of joint morphology and position [23]. Due to differences in craniofacial size, bite force and facial muscle strength [33], sex may also influence the characteristics of the TMJ [10, 26, 34]. A greater prevalence of TMD was observed in females [35] and in skeletal class II patients [8], which overlaps with the sample population in this study.

Imaging is one of the necessary auxiliary methods for diagnosing the internal conditions of the TMJ. Currently, CBCT is considered the most cost-effective method for determining linear and volumetric measurements of TMJ bone structure, with a short scan time, low radiation dose and low cost [36], as well as the ability to reflect bone damage [5] from multiplanar images without overlap, amplification or distortion [18]. In the standing position, the diagnostic error of the condyle position is reduced since the head is in a more physiological position [37].

**Fig. 7:**
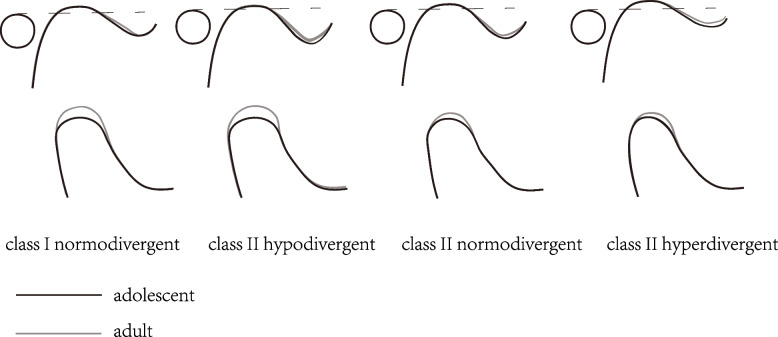
Changes in different skeletal patterns of the TMJ with age

The condyle is an important growth site of the mandible with a long growth period that can last up to the age of 20 years [12], and it retains its lifelong remodelling capability [14]. Even in adults, age also has an impact on size, shape, position and other aspects of the TMJ area [38]. The overall trend of changes in the TMJ with age in this study is summarized in Fig. 7. The final fit between the fossa and the condyle was likely to improve from adolescents to adults as the size of the condyle, including height, length, and long and short diameters increased while the glenoid fossa width and depth decreased. The mean differences in condylar height and length between the adults and adolescents of skeletal class I normodivergent and class II hypodivergent were approximately 2 mm, while those of class II normodivergent and hyperdivergent were only approximately 1 mm. This may be the reason why significant differences were not detected in condylar length and height among adolescents with different skeletal patterns but were detected in adults. According to Bjork’s study [12], there was a peak in condyle growth in males at approximately 14 years of age, with an average increase of approximately 5.5 mm/year along the growth direction, and there was no correlation between condyle length increment and growth direction. The difference between the results of this study and those of Bjork’s study was related to the measurement method. We made a progress in exploring the growth and development of condyles in specific skeletal class II patients with different vertical facial patterns in three dimensions since Bjork’s study explained the overall tendency and extrema of mandibular growth using a sample comprising patients with various types of malocclusions and craniofacial deformities [12] and demonstrated the growth of only one class II malocclusion patient with average facial height [13] using lateral cephalometry, although we used multiple-cross-sectional samples. The greatest condylar length and height differences between two ages were observed in skeletal class II hypodivergent patients, which was consistent with Bjork’s [12] conclusion that people with a low mandibular angle have more vertical condyle growth. In addition, the average differences in condylar long- and short-axis diameters of the two ages as well as the average values were also the greatest in class II hypodivergent patients. In adults, the mean condylar long- and short-axis diameters in class I normodivergent patients were approximately the same as those in class II normodivergent patients, while the condylar height and length were greater. However, significant differences in condylar height, length, and long and short diameters were found among adults with different vertical facial patterns. Loiola et al. [39]. compared the condylar volume and surface of 55 adult individuals with different sagittal skeletal patterns and found no significantly differences among skeletal class I, II and III patients. Similarly, a study carried out by Ceratti et al. [40]. revealed that condylar head volume was negatively associated with vertical patterns but no associations with skeletal class I, II or III were detected. It seemed that the effect of the sagittal skeletal pattern on the condyle was relatively limited compared to that of the vertical skeletal pattern. The condyle is a main component of the TMJ, and its shape and volume play important roles in the long-term stability of treatment outcomes for prosthetics, orthodontics and orthognathics [5, 41]. A large condyle can better match the glenoid fossa, which provides much more stable support when the bite force changes and better resists the occurrence of displacement than a small condyle [17, 42], thus reducing the subsequent dislocation of the disc [43]. In addition, patients with TMD tend to have smaller condyles [44], which are also associated with TMJ pain [45]. Therefore, the condyles of class II hypodivergent and class I normodivergent patients, with a greater length, height and diameter, seem to be more stable than those of class II normodivergent and hyperdivergent patients.

According to Katsavrias [46], the height of the articular eminence increases rapidly before the age of 7 and remains nearly unchanged between the ages of 7 and 11, and the remaining height is reached before the age of 20. The articular eminence inclination reaches its peak between the ages of 21 and 30 and decreases after the age of 31 [47], although some studies have shown that there is no significant difference among adults of different ages [48]. In this study, the glenoid fossa depth and articular eminence inclination decreased in adults compared with adolescents, suggesting that adaptive remodelling rather than growth dominated the development of the articular eminence between the ages of 14 and 18. Some researchers believe that the glenoid fossa depth and articular eminence inclination are closely related to the condylar morphology and position and mandibular functional movement [4, 28, 49]. However, whether they can increase the risk of TMD [25, 27] is still controversial [50]. Insufficient glenoid fossa depth may facilitate the crossing of the articular disc to the lowest point of the articular eminence during motion, which significantly increases the risk of dislocation of the articular disc [51]. Hence, for patients with small condyles and shallow glenoid fossae, such as class II hyperdivergent patients, special attention should be given before orthodontic treatment begins [3]. In addition, the normal range of the articular eminence inclination is 30°-60°; patients may present with intraarticular disorders outside this range [52]. Statistical differences in glenoid fossa depth and articular eminence inclination were found among different class II vertical skeletal patterns but not between class I and II normodivergent patients, similar to the results of Lin et al. [19], indicating that these differences might be one of the factors affecting the vertical growth and development of the mandible [53]. A shallow and flat articular fossa, a feature of class II hyperdivergent patients, has insufficient control of the condyle, and is therefore more likely to rotate clockwise during the period of growth and development, resulting in backwards and downwards growth trends of the mandible, thus forming the hyperdivergent facial type. In contrast, a deep and steep articular fossa, a feature of class II hypodivergent patients, forces the mandible to exhibit a counterclockwise growth trend, resulting in the formation of the hypodivergent facial type. Due to the lifelong remodelling ability of the TMJ, it cannot be ruled out that the formation of the glenoid fossa is related to the different bite forces and muscle contraction directions of patients with different vertical facial types. Hypodivergent patients had more powerful chewing muscles and a greater possibility of manifesting a deep overbite, which demanded a vertically overdeveloped articular eminence to satisfy a larger occlusal space during mouth opening [54]. However, the glenoid fossa width is likely not related to skeletal facial type [3, 19].

The increase in the condyle neck inclination and condyle head angle with age may be related to the average upwards and slight forwards growth direction of the condyle [12]. No significant difference was detected among different skeletal patterns within the same age group, suggesting that the skeletal patterns mainly interact with condylar size rather than shape. Ricketts believed that the mandibular arc could reflect mandibular or condylar rotation during growth, and it is increased approximately 3° every 5 years until the end of growth and development [55]. In this study, the mandibular arc also increased between the two age groups, with the change amplitudes of class II patients being approximately 3°, while it was only approximately 2° of class I normodivergent patients, indicating that counterclockwise rotation was common in the population with asymptomatic TMJs and was probably more obvious in class II patients. We speculated that this might be due to the compensatory mechanism arising from the less coordinated development of the upper and lower jaws in class II patients than in class I patients. The change amplitudes of the three class II vertical skeletal patterns were similar, and the largest average value of the mandibular arc was found in hypodivergent patients, while the smallest value was found in hyperdivergent patients in both adults and adolescents, suggesting that the formation of the vertical facial type was determined at an earlier stage and that adolescents exhibited a similar degree of rotational growth.

Similar to the results of Chae et al. [10], the position of the condyle and fossa remained basically stable during growth and development, with almost all the measurements showing no statistically differences between the two age groups. The fossa position may be an important factor in the occurrence of malocclusion since it determines the position of the condyle, changing the posterior facial height. The vertical position seemed to be the main interaction factor with the vertical facial type, as the transverse and anteroposterior positions showed no significant differences among the groups, consistent with the findings of a previous study [56]. In both age groups, hyperdivergent patients exhibited the highest fossa position, and hypodivergent patients exhibited the lowest fossa position. The condylar position is affected by many dynamic factors, such as growth and development, functional movement, occlusion, and TMJ remodelling [57]. The relationship between the skeletal pattern and condylar position (or joint space) is still controversial [18]. Most studies reported that patients with a skeletal class II facial type always presented reduced anterior [23, 25, 27] and superior [25] joint spaces, and patients with a hyperdivergent facial type manifested reduced superior and posterior joint spaces [3, 10, 19, 24]. In this study, no significant differences in anterior or posterior joint space were detected among the groups. The superior and lateral joint spaces were significantly larger in skeletal class II adults than in controls. Among the different vertical facial types, the smallest superior, mesial and lateral joint spaces were found in hyperdivergent patients. The clinical significance of the joint space and condylar position remains disputed [57, 58]. This study suggested that instead of a particular value, the sagittal condylar position, defined by the formula ln(P/A), as well as the joint space might have a spectrum of normal ranges within which the adaptive capacity could maintain the balance of the stomatognathic system and the normal function of the TMJ. However, the position and relationships of the condyle and fossa remained relatively constant during growth and development for every skeletal pattern, which is likely an important basis for TMJ stability and normal function.

There were some limitations in this study. First, some asymptomatic patients with internal TMJ derangement may have been recruited because the status of the articular disc could not be confirmed by CBCT. Second, the mandibular plane angle, which is defined as the inferior border of the angle and menton, tends to slightly decrease by approximately 1° every 3 years until 24 years of age for males [55], which may cause minor errors since we used two age groups with the same cephalometric value. A true longitudinal sample was optimal but difficult to realize due to ethical considerations. Third, this study was limited to females, and the findings may not be generalizable to males. The TMJ physiological and degenerative changes in the TMJ with age after 35 years of age were not included. In future studies, magnetic resonance imaging could be used to further confirm soft tissue conditions, such as those of the articular disc. Longitudinal studies are still needed to clarify whether changes in the structure and position of the TMJ and craniomaxillofacial structures are the causes of TMJ disorders. The TMJ characteristics of skeletal class II patients with various vertical facial patterns identified in this article can be used in orthodontic treatment planning and provide a theoretical basis for the establishment of relevant mechanical models, such as finite element analysis, to explore stress distribution in the TMJ region.

## Conclusion

1. The vertical skeletal pattern, rather than the class II sagittal skeletal pattern, may be the main factor affecting the morphology and position of the TMJ.
2. Class II hypodivergent patients presented the largest condylar long and short axes, the largest mandibular arc, the deepest glenoid fossa depth, the steepest articular eminence inclination, and the lowest glenoid fossa vertical position. The manifestations of class II hypodivergent patients were mostly the opposite.
3. The TMJ differences between patients 11–14 years old and patients 18–35 years old were mainly in the morphology rather than the position.

## Data Availability

The datasets used and/or analyzed during the current study are available from the corresponding author on reasonable request.

## Abbreviations

TMJ: Temporomandibular joint
CBCT: Cone-beam computed tomography
